# Longitudinal maternal glycemia during pregnancy and placental epigenetic age acceleration

**DOI:** 10.1186/s13148-025-01825-z

**Published:** 2025-02-07

**Authors:** Tesfa Dejenie Habtewold, Prabhavi Wijesiriwardhana, Richard J. Biedrzycki, Cuilin Zhang, Katherine L. Grantz, Jagteshwar Grewal, Fasil Tekola-Ayele

**Affiliations:** 1 Epidemiology Branch, Division of Population Health Research, Division of Intramural Research, Eunice Kennedy Shriver National Institute of Child Health and Human Development, National Institutes of Health, 6710B Rockledge Drive, Room 3204, Bethesda, MD 20892-7004 USA; 2 Glotech, Inc., Contractor for Division of Population Health Research, Division of Intramural Research, Eunice Kennedy Shriver National Institute of Child Health and Human Development, National Institutes of Health, Bethesda, MD USA; 3 Global Centre for Asian Women’s Health, Yong Loo Lin School of Medicine, National University of Singapore, Singapore, Singapore; 4 Bia-Echo Asia Centre for Reproductive Longevity and Equality (ACRLE), Yong Loo Lin School of Medicine, National University of Singapore, Singapore, Singapore; 5 Department of Obstetrics and Gynecology, Yong Loo Lin School of Medicine, National University of Singapore, Singapore, Singapore; 6 Department of Nutrition, Harvard T.H. Chan School of Public Health, Boston, MA USA

**Keywords:** Placental age acceleration, Placental aging, Hyperglycemia, Glycemic biomarkers, Pregnancy, Longitudinal modeling, Glucose, HbA1c, Insulin

## Abstract

**Background:**

Dysregulation of maternal glucose homeostasis has been related to an increased risk of morbidity and mortality in mothers and fetuses, yet the mechanism remains unclear. This study investigated the association between maternal glycemic levels and placental epigenetic age acceleration (PAA) in a multiethnic cohort.

**Methods:**

In a sample of 301 pregnant women (102 Hispanic, 77 White, 72 Black, and 50 Asian/Pacific Islander), the association of glycemic markers cumulative exposure with PAA was tested using linear regression adjusting for fetal sex, maternal age, educational status, and health insurance status. Models were applied in the full cohort and stratified by race/ethnicity. Further, sensitivity analyses were performed after excluding women with GDM or preeclampsia.

**Results:**

Among Black women, high glucose, HbA1c, and insulin cumulative exposure levels were associated with lower PAA compared to low cumulative exposure levels (*β* = − 0.75 weeks, 95% CI = − 1.41 to − 0.08); *β* = − 0.86, 95% CI = − 1.51 to − 0.21; and *β* = − 0.76, 95% CI = − 1.49 to − 0.03, respectively). Among Asian/Pacific Islander women, medium insulin cumulative exposure level was associated with lower PAA (*β* = − 0.94 weeks, 95% CI = − 1.74 to − 0.14). No significant association was observed among White and Hispanic women as well as in the full cohort.

**Conclusions:**

Elevated glucose, HbA1c, and insulin cumulative levels throughout pregnancy were associated with lower PAA in Black and Asian/Pacific Islander women. Placental epigenetic aging may be altered by maternal elevated glycemia and may in part underlie early programming of health outcomes in pregnancy and childhood health outcomes.

**Supplementary Information:**

The online version contains supplementary material available at 10.1186/s13148-025-01825-z.

## Background

Glucose homeostasis throughout pregnancy is critical for both maternal and fetal health [1]. Dysregulation of this physiological process results in complications with a large clinical and public health burden [1, 2]. Hyperglycemia is associated with an increased risk of perinatal complications [1, 3–6] and cardiometabolic disorders in later life in the mother and child [1, 7], yet the mechanism remains unclear. Understanding the link between maternal glycemic trajectory during pregnancy and molecular alterations in the maternal–placenta–fetal interface can potentially inform innovative interventions to avert the clinical consequences of glycemic dysregulation.

During pregnancy, the placenta plays a critical role in orchestrating the glycemic state of the maternal–fetal unit via glucose transportation, lipid sequestration, metabolic waste exchange, and hormone synthesis [8]. Meanwhile, the functions of the placenta are influenced by maternal metabolic, endocrine, and inflammatory environment [9]. Higher glycemic levels during pregnancy have been linked to placental developmental immaturity, placental aging-related molecular processes such as inflammation, oxidative stress, apoptosis, and epigenetic modifications [10–13]. Immaturity of placental villi has been consistently observed by gross morphology, histology, and ultrasound examinations among women with gestational diabetes mellitus (GDM) [10, 14]. Accelerated placental aging is associated with an increased risk of pregnancy complications that could be accompanied by poor childhood and adult health outcomes [15–18]. Therefore, maternal glycemic dysregulation during pregnancy may be linked to health outcomes through a potential influence on placental aging.

Molecular estimation of placental aging has been facilitated by recent developments in epigenetic clocks [15, 19]. Placental aging acceleration (PAA) indicates the epigenetic (biological) age of the placenta relative to its gestational age [15, 19]. Altered placental DNA methylation is commonly seen in pregnancies with elevated maternal blood glucose levels and GDM [20–22]. Similarly, changes in cord and child blood DNA methylation have been linked with cumulative glycemic exposure and glycemic trajectories across gestation [4, 5, 23, 24]. However, it is unknown whether cumulative glycemic exposure level and glycemic trajectory across gestation are associated with PAA. There is also a lack of data on the relationship between glycemic levels and PAA by maternal race/ethnicity, despite differences in glucose homeostasis [25–27] and placental phenotypes [28–31] across race/ethnic groups.

This study aimed to investigate the association between maternal glycemic markers (glycated hemoglobin (HbA1c), glucose, and insulin) longitudinally measured at four research visits during pregnancy and PAA in a racially/ethnically diverse cohort. Specifically, we investigated whether a glycemic marker’s cumulative exposure level across gestation is associated with PAA in the full cohort and stratified by race/ethnicity. In secondary analysis, we tested associations of a glycemic marker’s level per visit with PAA and a glycemic marker’s longitudinal trajectory with PAA.

## Methods

### Study population

Data from the *Eunice Kennedy Shriver* National Institute of Child Health and Human Development (NICHD) Fetal Growth Studies—Singleton's cohort were used [32]. The NICHD Fetal Growth Studies were a racially/ethnically diverse prospective pregnancy cohort study conducted between July 2009 and January 2013 at 12 clinical sites in the USA. A total of 2802 women with singleton pregnancies were recruited for the study, of which 2489 were excluded in the current study as they did not provide placenta samples. During the genotype quality control process, 11 women were excluded due to sex mismatch (*n* = 4) or outlier samples (*n* = 7), and one additional woman was excluded due to sample identifier mismatch. This resulted in a final sample of 301 women with good quality data for the main analysis [32, 33]. For the sensitivity analysis, 13 women with gestational diabetes mellitus and preeclampsia were further excluded. Consequently, the main analysis included 301 women (102 Hispanic, 77 White, 72 Black, and 50 Asian/Pacific Islander), while the sensitivity analysis focused on a reduced sample of 288 women (Figure S1). The inclusion and exclusion criteria, ethical approval, and data collection, and quality control process have been described previously [32]. Pregnant women completed sociodemographic, reproductive, and pregnancy history questionnaires at enrollment. Gestational age at delivery (in weeks) was determined using the date of the last menstrual period and confirmed by ultrasound between 8^+0^ and 13^+6^ weeks of gestation. Additional medical data were extracted from medical records.

### Measurement of glycemic markers

**Fig. 1:**
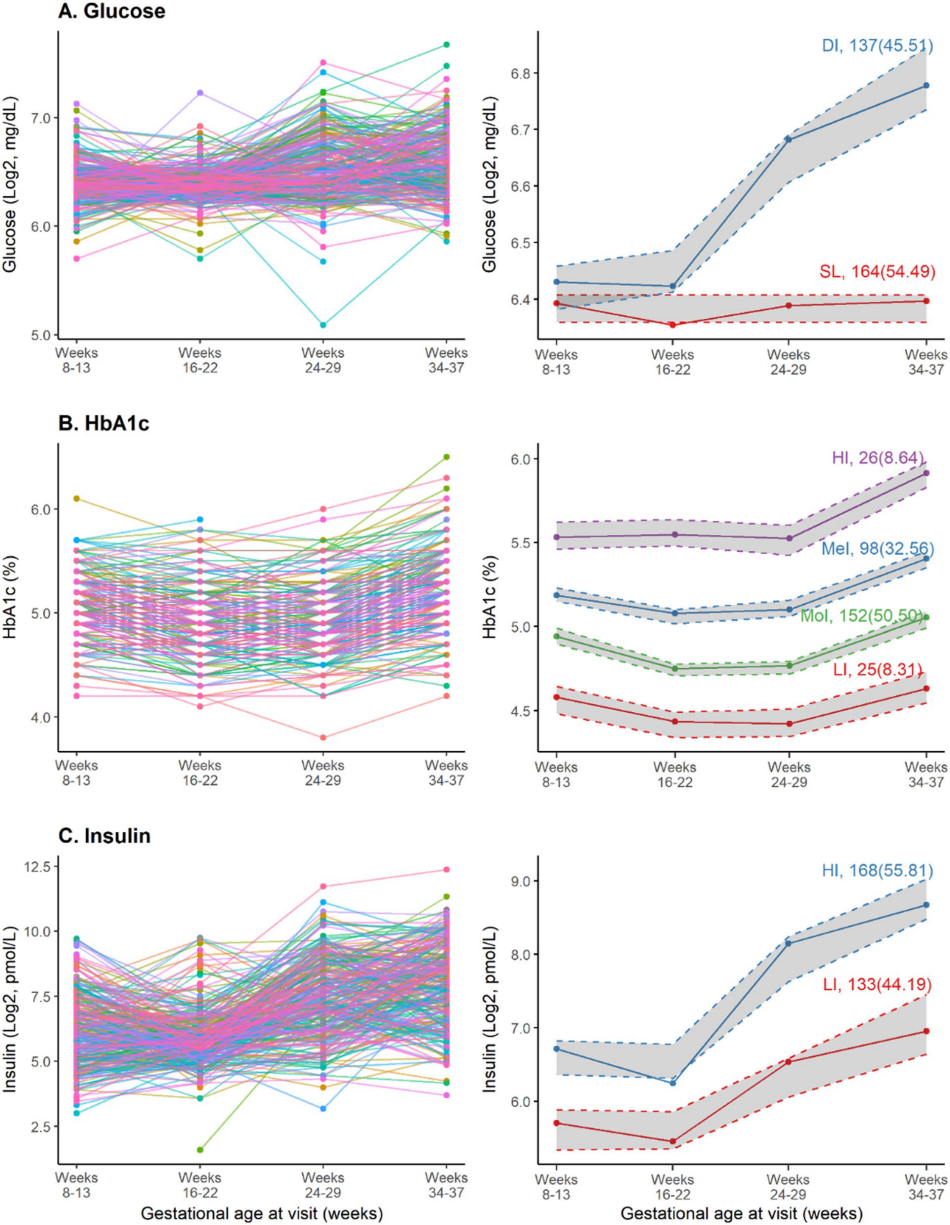
Spaghetti plot (left panel) and trajectories (right panel) of maternal glucose (**A**), HbA1c (**B**), and insulin (**C**). Different colors in the spaghetti plot represent individual woman. Solid lines in the trajectory plots denote mean glycemic trajectory for a respective trajectory group. Shaded gray areas and broken lines indicate upper and lower bounds of 95% confidence interval (CI). DI = dramatically increasing; HI = high increasing; LI = low increasing; MeI = medium increasing; MoI = moderate increasing; SL = stably low

Maternal blood samples were collected at 8–13 (enrollment), 16–22 (visit 1, fasting), 24–29 (visit 3), and 34–37 (visit 4) gestational weeks (Fig. 1). Samples were processed immediately after collection and stored at − 80 °C. Glycemic markers were measured at the University of Minnesota Department of Laboratory Medicine and Pathology. Plasma insulin (pmol/L) and glucose (mg/dL) were measured with electrochemiluminescence immunoassay-sandwich principle (Roche Diagnostics, Indianapolis, Indiana, USA) and enzymatic method (Roche Diagnostics, Indianapolis, Indiana, USA). Glycated hemoglobin (HbA1c, %) was measured using red blood cells with a non-porous ion exchange High-Performance Liquid Chromatography (HPLC) assay (Tosoh Automated Analyzer HLC-723G8, Tosoh Bioscience, Inc., South San Francisco, CA & Tokyo, Japan). The intra-assay CVs (i.e., a measure of the variance between data points within an assay) at the lowest and highest detectable biomarker levels were 3.1% and 4.2% for insulin, 2.4% and 3.1% for glucose, and 1.2% and 0.6% for HbA1c, respectively. Raw data were standardized (z-score) or log_2_-transformed before analyses to fulfill normality assumptions.

### Placental age acceleration (PAA)

Placental parenchymal biopsies measuring 0.5 cm × 0.5 cm × 0.5 cm were taken within one hour of delivery directly below the fetal side of the placenta and placed in RNALater and frozen for molecular analysis. The placental biopsies were handled and processed at Columbia University Irving Medical Center [34]. DNA was assayed for methylation using Illumina’s Infinium Human Methylation450 Beadchip (Illumina Inc., San Diego, CA) following standard Illumina protocols for background correction, normalization to internal control probes, and quantile normalization [33]. Details on DNA methylation data processing and quality control were described in a previous study [33]. After quality control, methylation data at 409,101 cytosine‐phosphate‐guanine sites (CpGs) were analyzed.

We estimated placental epigenetic age using the refined robust placental clock (rRPC) developed using 395 CpGs for uncomplicated term pregnancies [19]. PAA was the residual of the placental epigenetic age regressed on gestational age at delivery and was used as a measure of the biological age of the placenta [33].

### Statistical analyses

Characteristics of the study participants were summarized using frequency (%) for categorical variables and mean (SD) for continuous variables. The difference in these characteristics was compared across the race/ethnic groups using analysis of variance (ANOVA) or Kruskal–Wallis test for continuous variables and Chi-squared or Fisher’s exact test for the categorical variables.

#### Association between cumulative glycemic exposure and PAA

Maternal cumulative glycemic concentration during pregnancy was estimated using area under the glycemic marker concentration versus time curve (AUC) analyses [35, 36]. First, linear mixed model was fitted using log_2_-transformed value of measurements at enrollment, visit 1, visit 3, and visit 4 to estimate the predicted glycemic marker levels with varying intercept and varying linear and quadratic slopes for gestation age at each visit per individual. Then, AUC_gluc_ (mg/dL*week), AUC_HbA1c_ (%*week), and AUC_insl_ (pmol/L*week) were separately calculated using the following integral formula: ${\int }_{0}^{t}f\left({x}_{i}\right)= {\beta }_{0i}+{\beta }_{1i}{x}_{i}+{\beta }_{2i}{x}_{i}^{2}$, where *t* is gestational age at delivery, *β*_0_ is the random effects intercept, *β*_1_ is the random effects linear slope for gestational age at visit, *β*_2_ is the random effects quadratic slope for gestational age at visit, and *x* is gestational age at the visit for each individual *i*. Integrals were calculated using the “integrate” function of the *stats* R package. The calculated AUC was then grouped into tertiles, with tertiles 1, 2, and 3 representing *low*, *medium*, and *high* cumulative exposure of glycemic markers (Table S1), respectively. Finally, linear regression analyses were performed to test whether medium and high cumulative exposures were associated with PAA compared to low cumulative exposure. Models were adjusted for fetal sex (categorical), maternal age (continuous), maternal educational status (categorical), and health insurance ownership (categorical). A two-sided *p* < 0.05 was taken as evidence for a statistically significant association.

#### Association between glycemic levels at each gestational visit and PAA

To examine whether glycemic alteration is associated with PAA at a specific gestational age window, linear regression analyses were performed between HbA1c (*Z*-score, %), glucose (*Z*-score, mg/dL), and insulin (log_2_, pmol/L) and PAA at each of the four study visits (i.e., 8–13, 16–22, 24–29, and 34–37 weeks). Models were adjusted for gestational week at visit in addition to the above-mentioned covariates.

#### Association between trajectories of glycemic markers and PAA

Group-based trajectory modeling (GBTM) was performed using PROC TRAJ SAS macro to identify distinct subgroups of pregnant women with unique glycemic profiles and assess their gestational trajectories [37]. Gestational visits (in weeks) were coded as a time variable and glycemic markers as an outcome variable—glucose (log_2_, mg/dL), insulin (log_2_, pmol/L), and HbA1c (%). We started with one group and repeatedly increased the number of groups with a quadratic polynomial order until a model with an optimum number of trajectory groups was identified. The optimal number of trajectory groups was selected based on sample size-adjusted Bayesian information criterion (BICn) and log Bayes factor (i.e., 2*ΔBICn) (Table S2). ΔBICn was calculated by subtracting the absolute value of BICn of the less complex model from the absolute value of BICn of the more complex model. The model with the lowest BICn, log Bayes factor > 10, and at least 20 individuals per group was selected as the best-fitting model. Then, the polynomial order was adjusted until it became significant at the significance level (α) of 0.05 for all trajectory groups. The drop-out model, which includes a logistic model of drop-out probability per gestational visit, was used to investigate whether the attrition rates significantly biased group membership probabilities of trajectories [38]. For each group, we assumed that the drop-out probability depends on the previous measures. A model with an average group posterior probability of 70% and above is considered accurate in classifying individuals [39]. The classification accuracies of our models were 85 to 95%, indicating good model accuracy in the classification of individuals into the respective trajectory groups based on glycemic levels. Linear regression analyses were performed to test the association between a glycemic biomarker group and PAA using trajectory groups with low glycemic levels as a reference, adjusted for covariates mentioned above.

Models were applied in the full cohort and stratified by race/ethnicity. Considering that women with and without GDM and preeclampsia differ in glycemic trajectories and PAA, sensitivity analyses were performed after excluding 13 women who developed GDM or preeclampsia during the current pregnancy.

All data analyses were performed in R statistical software version 4.3.2 unless otherwise specified.

## Results

### Characteristics of study participants

The majority (77.4%) of women were married or living with a partner, and 69% had at least a high school diploma. The mean (± SD) HbA1c at 8–13, 16–22, 24–29, and 34–37 gestational weeks was 5.1 (± 0.3), 4.9 (± 0.3), 4.9 (± 0.3), and 5.2 (± 0.4) %, respectively. The mean (± SD) glucose at 8–13, 16–22, 24–29, and 34–37 gestational weeks was 85.8 (± 11.7), 84.2 (± 9.60), 94.0 (± 19.5), and 97.6 (± 20.8) mg/dL, respectively. The mean (± SD) insulin at 8–13 (enrollment), 16–22 (visit 1), 24–29 (visit 2), and 34–37 gestational weeks was 121 (± 143), 86.1 (± 118), 289 (± 354), and 418 (± 493) pmol/L, respectively. Cumulative exposure levels of all three glycemic markers and PAA did not significantly vary by race/ethnicity (Table 1).

**Table 1:** Distribution of major characteristics of the study population

Characteristics, *n* (%) or mean (± SD)	Overall (*N* = 301)	Self-reported race/ethnic groups				Group difference (*p* value)
		Non-Hispanic White (*N* = 77)	Non-Hispanic Black (*N* = 72)	Hispanic (*N* = 102)	Asian/Pacific Islander (*N* = 50)	
Age (years)	27.68 (± 5.26)	29.64 (± 3.93)	25.65 (± 5.75)	26.60 (± 5.54)	29.80 (± 3.92)	**< 0.001**
Born in the USA, yes	192 (63.79)	75 (97.40)	65 (90.28)	42 (41.18)	10 (20.00)	**< 0.001**
Marital status, married or living with partner	233 (77.41)	74 (96.11)	36 (50.00)	76 (74.51)	47 (94.00)	**< 0.001**
Educational status, greater than high school	208 (69.10)	72 (93.51)	42 (58.33)	55 (53.92)	39 (78.00)	**< 0.001**
Employment status, employed	246 (81.73)	69 (89.61)	50 (69.44)	86 (84.31)	41 (82.00)	**0.012**
Health insurance ownership, private or managed care	184 (61.13)	71 (92.21)	35 (48.61)	37 (36.27)	41 (82.00)	**< 0.001**
Gestational age at delivery (weeks)	39.48 (± 1.14)	39.45 (± 1.07)	39.61 (± 0.93)	39.46 (± 1.19)	39.37 (± 1.39)	0.605
Refined robust placental clock (rRPC)/PAA	0.00 (± 1.00)	− 0.01 (± 0.93)	0.05 (± 1.16)	0.04 (± 0.91)	− 0.15 (± 1.01)	0.544
HbA1c (%)						
Weeks 8–13	5.05 (± 0.30)	5.04 (± 0.26)	5.09 (± 0.33)	4.99 (± 0.30)	5.11 (± 0.26)	0.086
Weeks 16–22	4.91 (± 0.33)	4.84 (± 0.30)	4.94 (± 0.34)	4.91 (± 0.35)	4.99 (± 0.30)	0.115
Weeks 24–29	4.92 (± 0.33)	4.87 (± 0.33)	4.95 (± 0.34)	4.87 (± 0.29)	5.04 (± 0.37)	**0.039**
Weeks 34–37	5.22 (± 0.37)	5.12 (± 0.39)	5.23 (± 0.34)	5.21 (± 0.33)	5.38 (± 0.43)	**0.013**
Glucose (mg/dl)						
Weeks 8–13	85.79 (± 11.65)	86.50 (± 8.08)	82.60 (± 9.66)	85.69 (± 13.83)	89.33 (± 13.01)	**0.008**
Weeks 16–22	84.16 (± 9.60)	84.39 (± 7.53)	81.68 (± 9.13)	84.91 (± 11.26)	85.76 (± 8.94)	0.082
Weeks 24–29	94.03 (± 19.47)	97.89 (± 18.57)	91.67 (± 17.35)	90.69 (± 21.24)	98.08 (± 18.61)	**0.005**
Weeks 34–37	97.60 (± 20.84)	97.96 (± 17.16)	95.58 (± 18.92)	94.44 (± 19.70)	106.39 (± 27.80)	**0.039**
Insulin (*p*/mol)						
Weeks 8–13	121.49 (± 142.73)	92.57 (± 121.16)	141.68(± 150.43)	136.28 (± 167.75)	109.06 (± 96.93)	**0.028**
Weeks 16–22	86.09 (± 118.08)	52.84 (± 48.96)	98.38 (± 121.40)	107.94 (± 153.98)	74.67 (± 89.22)	**< 0.001**
Weeks 24–29	289.11 (± 353.90)	253.27 (± 256.19)	328.64(± 461.64)	297.94 (± 365.08)	271.18 (± 285.82)	0.865
Weeks 34–37	418.33 (± 493.01)	303.91 (± 283.04)	509.94(± 701.17)	405.49 (± 460.45)	482.36 (± 423.97)	**0.042**
HbA1c cumulative exposure levels						0.164
Low	101 (33.55)	32 (41.56)	21 (29.17)	34 (33.33)	14 (28.00)	
Medium	100 (33.22)	27 (35.06)	23 (31.94)	37 (36.27)	13 (26.00)	
High	100 (33.22)	18 (23.38)	28 (38.89)	31 (30.39)	23 (46.00)	
Glucose cumulative exposure levels						0.642
Low	101 (33.55)	25 (32.47)	23 (31.94)	40 (39.22)	13 (26.00)	
Medium	100 (33.22)	25 (32.47)	25 (34.72)	34 (33.33)	16 (32.00)	
High	100 (33.22)	27 (35.06)	24 (33.33)	28 (27.45)	21 (42.00)	
Insulin cumulative exposure levels						0.263
Low	101 (33.55)	31 (40.26)	18 (25.00)	39 (38.24)	13 (26.00)	
Medium	100 (33.22)	26 (33.77)	27 (37.50)	31 (30.39)	16 (32.00)	
High	100 (33.22)	20 (25.97)	27 (37.50)	32 (31.37)	21 (42.00)	
GDM or preeclampsia, yes	13 (4.32)	3 (3.90)	3 (4.17)	5 (4.90)	2 (4.00)	1.00
Fetal sex, female	149 (50%)	38 (49%)	39 (54%)	53 (52%)	19 (38%)	0.318

Bold indicates statistically significant results (*p* value < 0.05)

SD, standard deviation; HbA1c, glycated hemoglobin; GDM, gestational diabetes mellitus; +, to compare group difference, ANOVA or Kruskal–Wallis test was applied for continuous variables and Chi-squared or Fisher's exact test was applied for categorical variables; PAA, placental epigenetic age acceleration

### Cumulative glycemic exposure and PAA

**Fig. 2:**
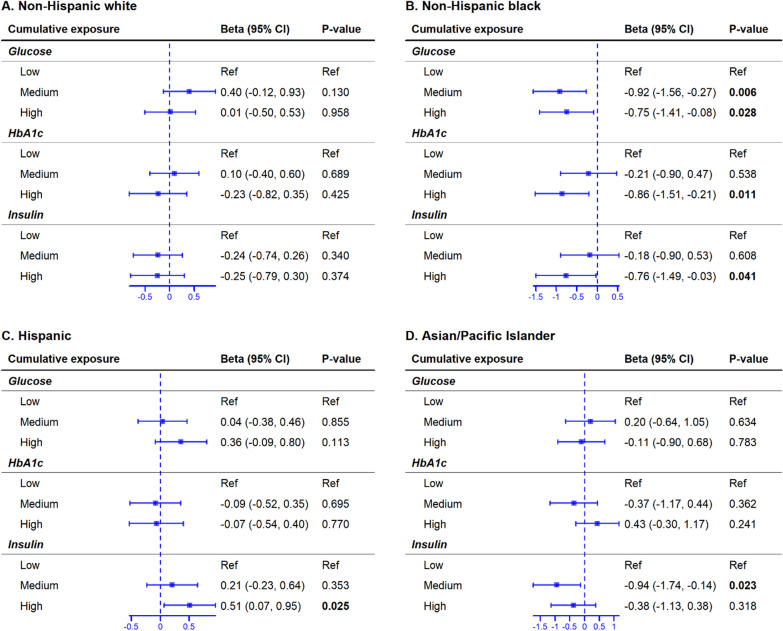
Change in PAA associated with cumulative glycemic marker exposure levels by race/ethnicity. Each horizontal line indicates the lower and upper bounds of the 95% CI, and a rectangle in the middle of the horizontal line indicates the average change (i.e., beta) in PAA. Broken vertical lines indicate the null hypothesis of no change. Low cumulative exposure is the reference group for each glycemic marker

In the full cohort, there were no cumulative glycemic marker levels significantly associated with PAA (Figure S2). Among Black women, high insulin, high HbA1c, and medium and high glucose cumulative exposure levels were associated with lower PAA compared to low cumulative exposure levels (*β* = 0.76 weeks, 95% CI = − 1.49 to − 0.03, *p* = 0.041; *β* = 0.86, 95% CI = − 1.51 to − 0.21, *p* = 0.011; *β* = 0.92, 95% CI = − 1.56 to − 0.27, *p* = 0.006; and *β* = 0.75, 95% CI = − 1.41 to − 0.08, *p* = 0.028, respectively). The associations, except for insulin, remained significant in sensitivity analyses excluding pregnancies complicated by GDM and preeclampsia (Fig. 2; Figure S3). Among Asian/Pacific Islander women, medium insulin cumulative exposure level was associated with 0.94 weeks lower PAA compared to low cumulative insulin exposure level (95% CI = − 1.74 to − 0.14, *p* = 0.023). All associations remained significant in sensitivity analyses (Fig. 2; Figure S3). In contrast, among Hispanic women, high insulin cumulative exposure level was associated with 0.51 weeks higher PAA compared to low cumulative exposure level (95% CI = 0.07 to 0.95, *p* = 0.025); however, the association was no longer significant in sensitivity analysis (Fig. 2; Figure S3).

### Glycemic levels at consecutive gestational age windows and PAA

**Fig. 3:**
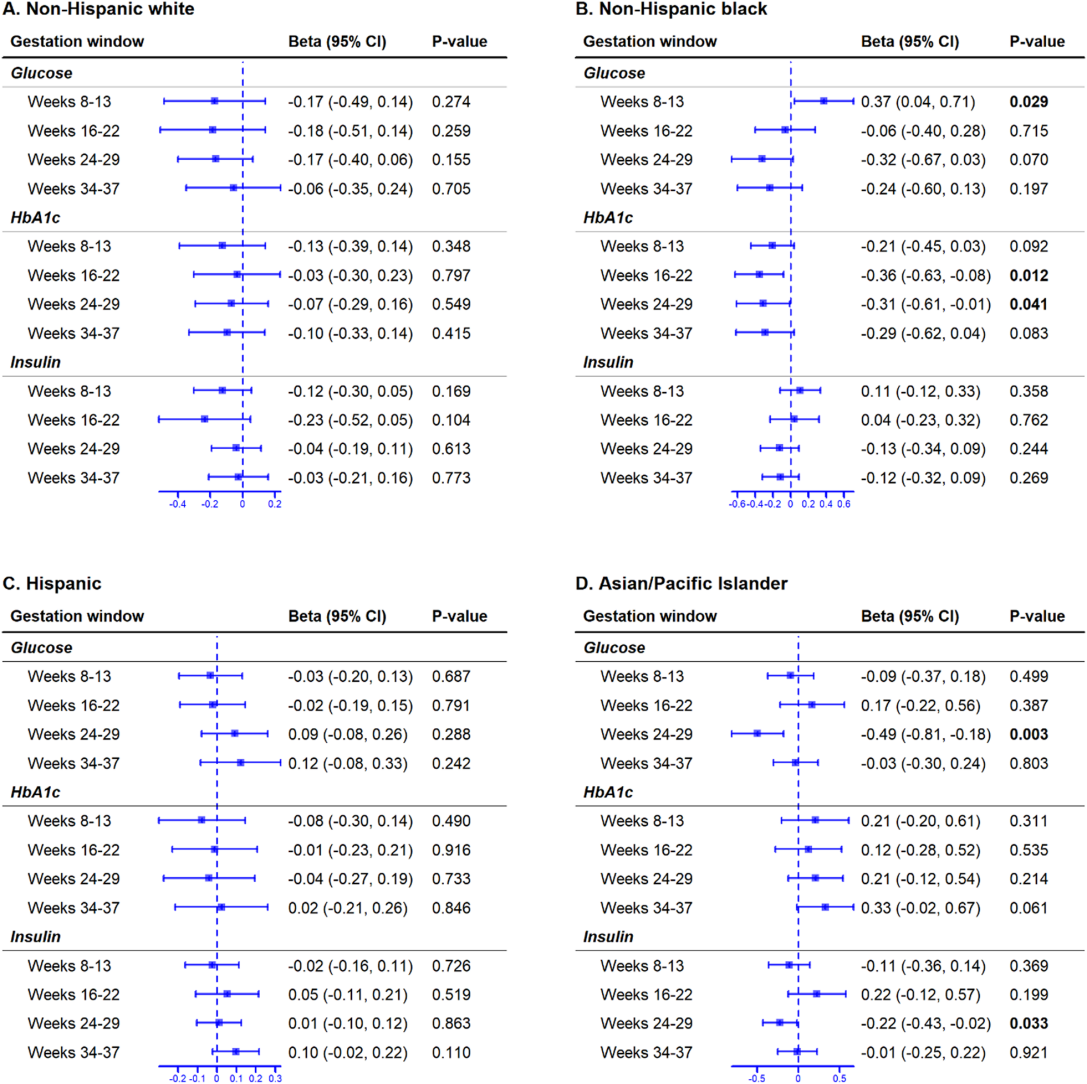
Change in PAA associated with glycemic marker changes at each gestational window by race/ethnicity. Each horizontal line indicates the lower and upper bounds of the 95% CI, and a rectangle in the middle of the horizontal line indicates the average change (i.e., beta) in PAA. Broken vertical lines indicate the null hypothesis of no change

Among Asian/Pacific Islander women, a 1 SD increase in glucose and a one-point increase in log_2_ insulin at weeks 24–29 were associated with lower PAA (*β* = 0.49 weeks, 95% CI = − 0.81 to − 0.18, *p* = 0.003 and *β* = 0.22, 95% CI = − 0.43 to − 0.02, *p* = 0.033, respectively). The associations remained significant in sensitivity analyses (Fig. 3; Figure S4). We observed that glucose levels at gestation weeks 24–29 in the full cohort and HbA1c at gestation weeks 16–22 and 24–29 among Black women were associated with lower PAA, but the associations did not persist in the sensitivity analyses (Fig. 3; Figure S5).

### Glycemic trajectories and PAA

We identified *stably low* (54.49%) and *dramatically increasing* (45.51%) glucose trajectories; *low increasing* (44.19%) and *high increasing* (55.81%) insulin trajectories; and four steadily increasing HbA1c trajectories—*low increasing* (8.31%), *medium increasing* (32.56%), *moderate increasing* (50.50%), and *high increasing* (8.64%) (Fig. 1; Table S3).

**Fig. 4:**
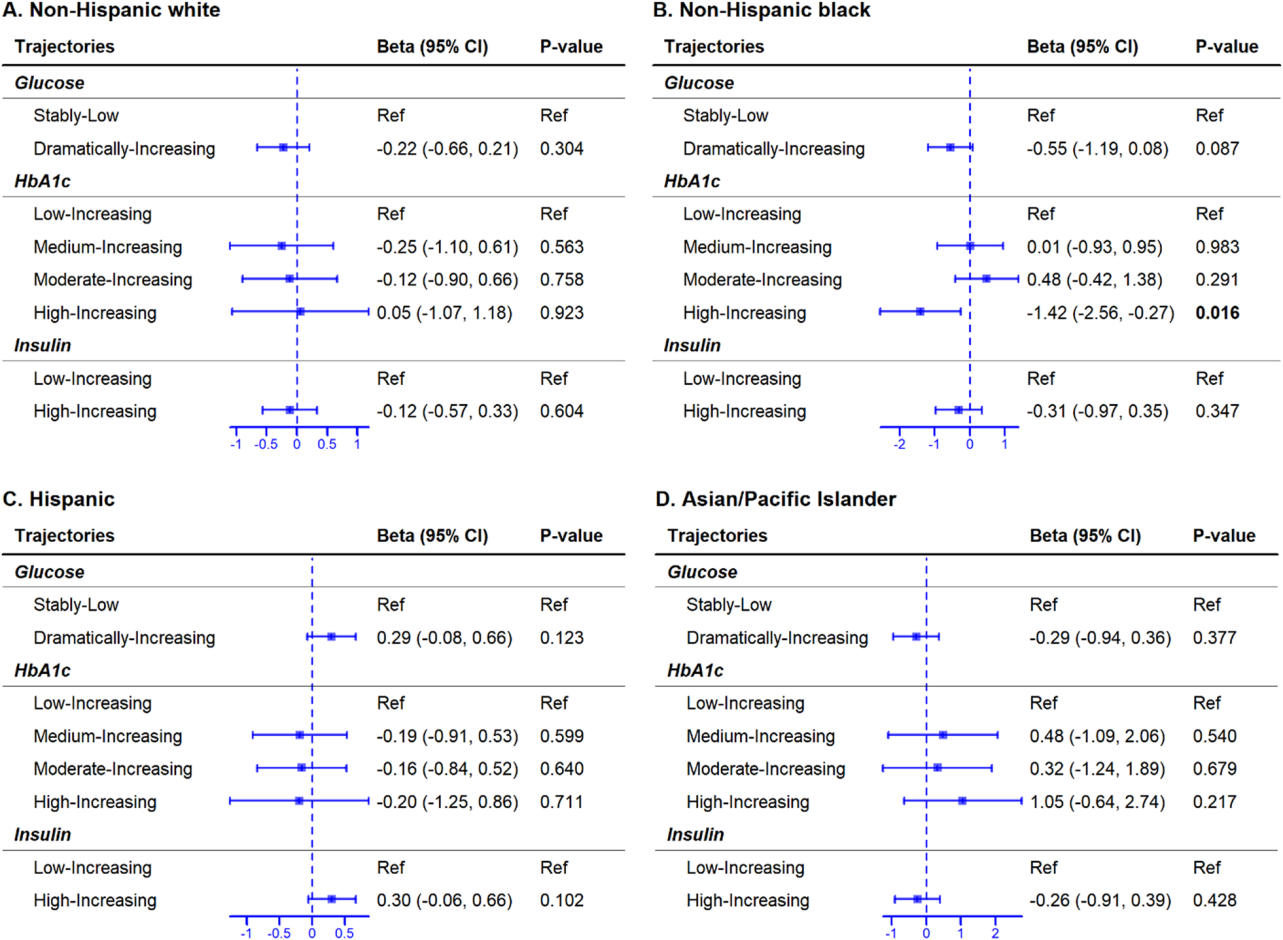
Change in PAA associated with glycemic marker trajectories. Each horizontal line indicates the lower and upper bounds of the 95% CI, and a rectangle in the middle of the horizontal line indicates the average change (i.e., beta) in PAA. Broken vertical lines indicate the null hypothesis of no change. Lowest order trajectory group is the reference group for each glycemic marker

In full cohort, trajectories of the three glycemic markers were not significantly associated with PAA (Figure S6). Among Black women, high increasing HbA1c trajectory was associated with 1.42 weeks lower PAA compared to low increasing HbA1c trajectory (95% CI = − 2.56 to − 0.27, *p* = 0.016), and the association remained significant in sensitivity analysis (Fig. 4; Figure S7).

## Discussion

In this study of longitudinally measured maternal glycemic markers during pregnancy, lower PAA was associated with higher glycemic markers in race/ethnic-specific analyses, while no associations were found in the full cohort. Specifically, 0.75–1.42 weeks lower PAA was observed among self-identified Black women in the medium or high cumulative glucose exposure group and in the high cumulative HbA1c exposure/trajectory group compared to Black women in the low glycemic marker group. Moreover, a 0.94-week lower PAA was found among Asian/Pacific Islander women in the medium cumulative insulin exposure group compared to those in the low exposure group, as well as a significantly lower PAA with increasing insulin and glucose in the late second trimester/early third trimester.

Previous studies have reported lower cord blood epigenetic age acceleration in pregnancies diagnosed with GDM [40] and lower neonatal blood epigenetic age acceleration in newborns of women with insulin-treated GDM in a previous pregnancy [41]. Similar studies in placenta are scarce, with some placental studies finding that higher maternal gestational weight gain has been associated with lower PAA [42], and low HDL cholesterol during early gestation has been associated with higher PAA [43]. During pregnancy, glycemic metabolism is increased, and a state of insulin resistance occurs in part through the influence of placental hormones to fulfill the energy demands of the mother and fetus [44]. When the placenta is exposed to high levels of glucose during pregnancy, elevated oxidative stress mechanisms impair its development and function [45] and lead to aging-related changes such as immaturity of chorionic villus [10] and epigenetic alterations [20]. Our finding of lower PAA in relation to elevated glycemic exposure is in line with histopathological and ultrasound observations of placental immaturity in hyperglycemic pregnancies [10, 14] and suggests that the delay in placental maturity may be explained by epigenetic molecular changes that impact placental development.

The cumulative glycemic marker measure used by our study reflects the extent of exposure of the placenta to glycemia throughout pregnancy [46]. The association of cumulative glycemic exposure with lower PAA was corroborated with a secondary analysis using the longitudinal trajectory of glycemic markers. Glycemic levels at individual visits yielded an association with lower PAA only in later gestation (weeks 24–29). The glycemic marker levels of the majority of our study participants are within the clinically normal range, which partly explains why the change in PAA associated with glycemia is due to cumulative elevated exposure, rather than a single visit’s elevated exposure. In agreement with this finding, other phenotypes such as maternal depression sustained across the first and second trimesters of pregnancy are associated with PAA [47].

The biological and clinical implications of placental epigenetic age deceleration are not as clear as that of acceleration. Maternal factors such as early gestation dyslipidemia and depression during pregnancy have been linked to higher PAA [43, 47], which in turn is associated with low birthweight [33] and preeclampsia [15]. Higher maternal gestational weight gain has been linked to lower PAA [42], similar to the elevated glycemia found in the present study. Epigenetic age deceleration in neonatal/cord blood at birth has been associated with delayed pubertal timing [48], lower weight from birth through 9 months [49], and slower growth in weight and BMI at age 7 and age 17 [50]. Based on these observations, it has been suggested that epigenetic age deceleration at birth or early childhood may indicate developmental immaturity [51]. In placenta, delayed villous maturation has been linked to fetal death [52] and delayed neurodevelopment in children [53], which have a high preponderance in hyperglycemic pregnancies [54, 55]. Therefore, the 0.75–1.5 weeks lower PAA found in Black and Asian women with high glycemic levels may indicate delayed developmental maturity of the placenta relative to its gestational age and is likely to be clinically important, but further studies are needed to strengthen this evidence.

The association between glycemic levels and PAA in our study was race/ethnic-specific. Previous studies have documented race/ethnic differences in glycemic levels and the distribution of placental pathologies in women with or without a known GDM diagnosis. Black and Asian pregnant women had higher HbA1c and serum glucose levels [25, 26], as well as lower insulin production and higher insulin resistance [27], compared to White women, regardless of diabetes status. Placental inflammatory lesions [28], maternal vascular malperfusion of placenta [31], low placental weight [28], and shorter placental telomere length [29] are also more common among Black women compared to White women. These differences in glycemic markers are also observed in the general population [56, 57], potentially due to underlying differences in socioeconomic status [58], nutritional factors [59], and access to health care [56]. Future studies in larger datasets are required to validate our findings and identify factors that may explain why the associations of glycemia with PAA are particularly specific to Black and Asian women.

A limitation of our study is the small sample size in each race/ethnic group, potentially limiting the study’s power to detect associations. Further larger studies are needed to assess whether absence of associations in some race/ethnic groups is because of limited statistical power. All placenta samples were obtained at delivery around 39 gestational weeks [33], limiting the study’s ability to estimate placental epigenetic age at different windows of gestation and make inferences on the association with the glycemic markers. Integrating the glycemia trajectories in to insulin resistance metrics could improve interpretation but could not be accomplished in the current study because longitudinal fasting blood samples were not available.

## Conclusions

Elevated glucose, HbA1c, and insulin cumulative levels throughout pregnancy in Black and Asian/Pacific Islander women, as well as at the end of the 2nd trimester, were associated with lower PAA. Even for clinically normal glycemic marker levels, elevated cumulative glycemic marker exposure was related to placental aging. Elevated glycemia-related placental epigenetic aging mechanisms may contribute to early programming of poor pregnancy and childhood health outcomes in relation to glycemic dysregulation during pregnancy.

## Supplementary Information

Additional file 1

Additional file 2

## Data Availability

The placental DNA methylation data are available through dbGaP with accession number phs001717.v1.p1. The analytic codes for the current study are available upon request to the corresponding author.

## Abbreviations

ANOVA: Analysis of variance
AUC: Area under the curve
AvePP: Average group posterior probability
BICn: Sample size-adjusted Bayesian information criterion
GDM: Gestational diabetes mellitus
DNA: Deoxyribonucleic acid
DNAm: Deoxyribonucleic acid methylation
GBTM: Group-based trajectory modeling
HbA1c: Glycated hemoglobin
HPLC: High-performance liquid chromatography
NICHD: National Institute of Child Health and Human Development
PAA: Placental age acceleration
PreE: Preeclampsia
rRPC: Refined robust placental clock
RNAs: Ribonucleic acids
SD: Standard deviation
