# Supplementary material for: Longitudinal maternal glycemia during pregnancy and placental epigenetic age acceleration
Source: Clin Epigenetics. 2025 Feb 7;17:19. doi: 10.1186/s13148-025-01825-z (PMC11803985; doi:10.1186/s13148-025-01825-z)

**Figure S1:** Sample selection flow chart.

**
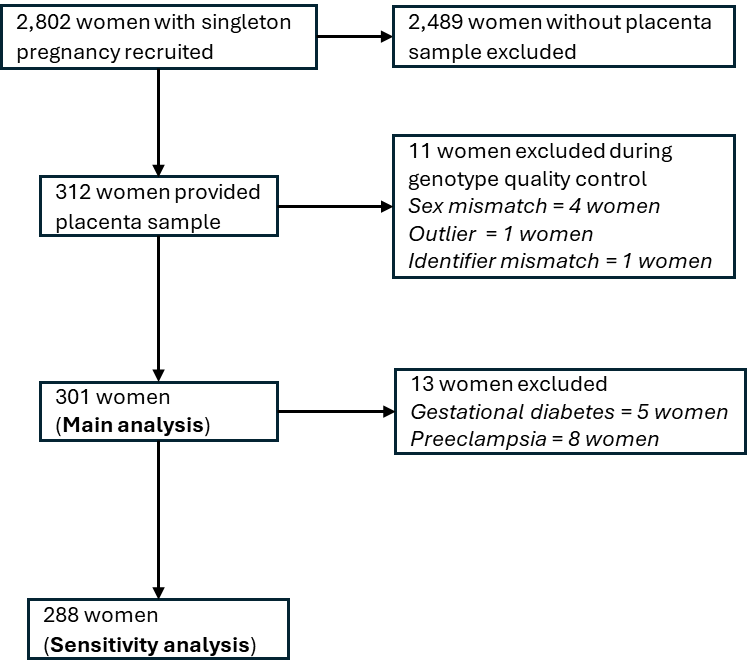
**

**Figure S2:** Change in PAA associated with cumulative glycemic marker exposure levels in the full cohort with (left panel) and without (right panel) GDM or preeclampsia (PreE). Low exposure level and low-order trajectory groups are reference groups. Each horizontal line indicates the lower and upper bounds of the 95% CI, and a rectangle in the middle of the horizonal line indicates the average change (i.e., beta) in PAA. Broken vertical lines indicate the null hypothesis of no change.


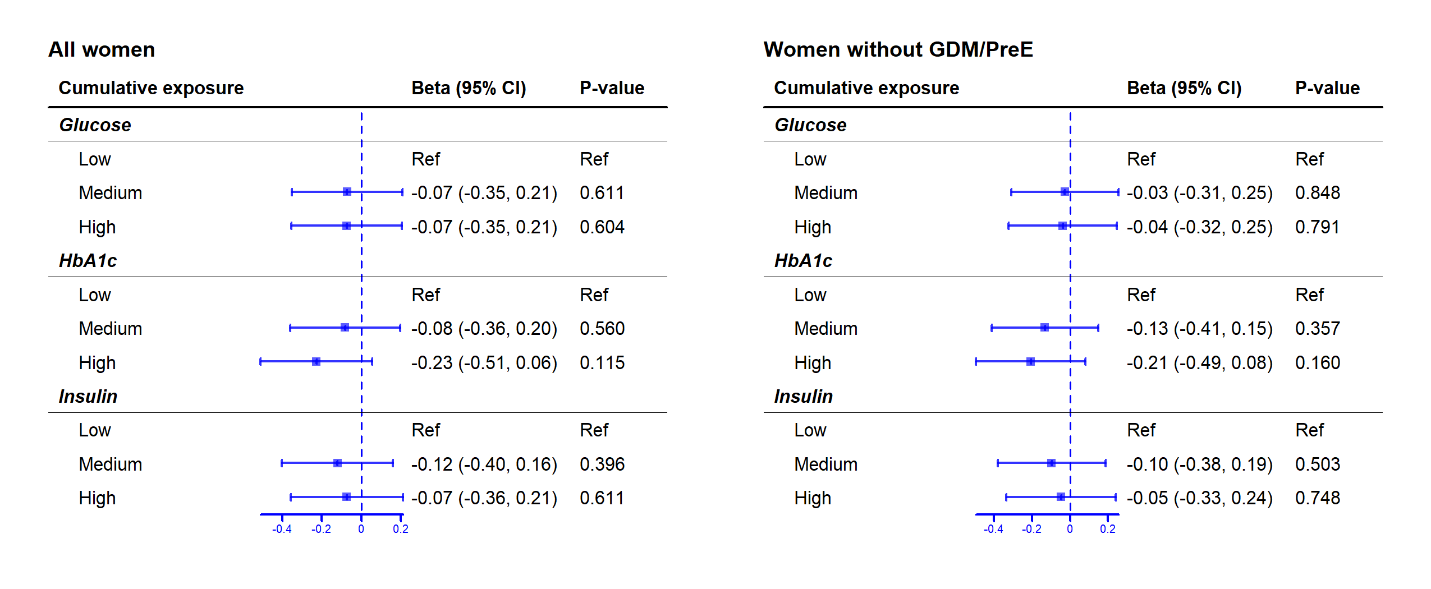


**Figure S3:** Change in PAA associated with cumulative glycemic marker exposure levels among women without GDM or preeclampsia by race/ethnicity. Each horizontal line indicates the lower and upper bounds of the 95% CI, and a rectangle in the middle of the horizonal line indicates the average change (i.e., beta) in PAA. Broken vertical lines indicate the null hypothesis of no change. Low exposure level is the reference group for each glycemic marker.


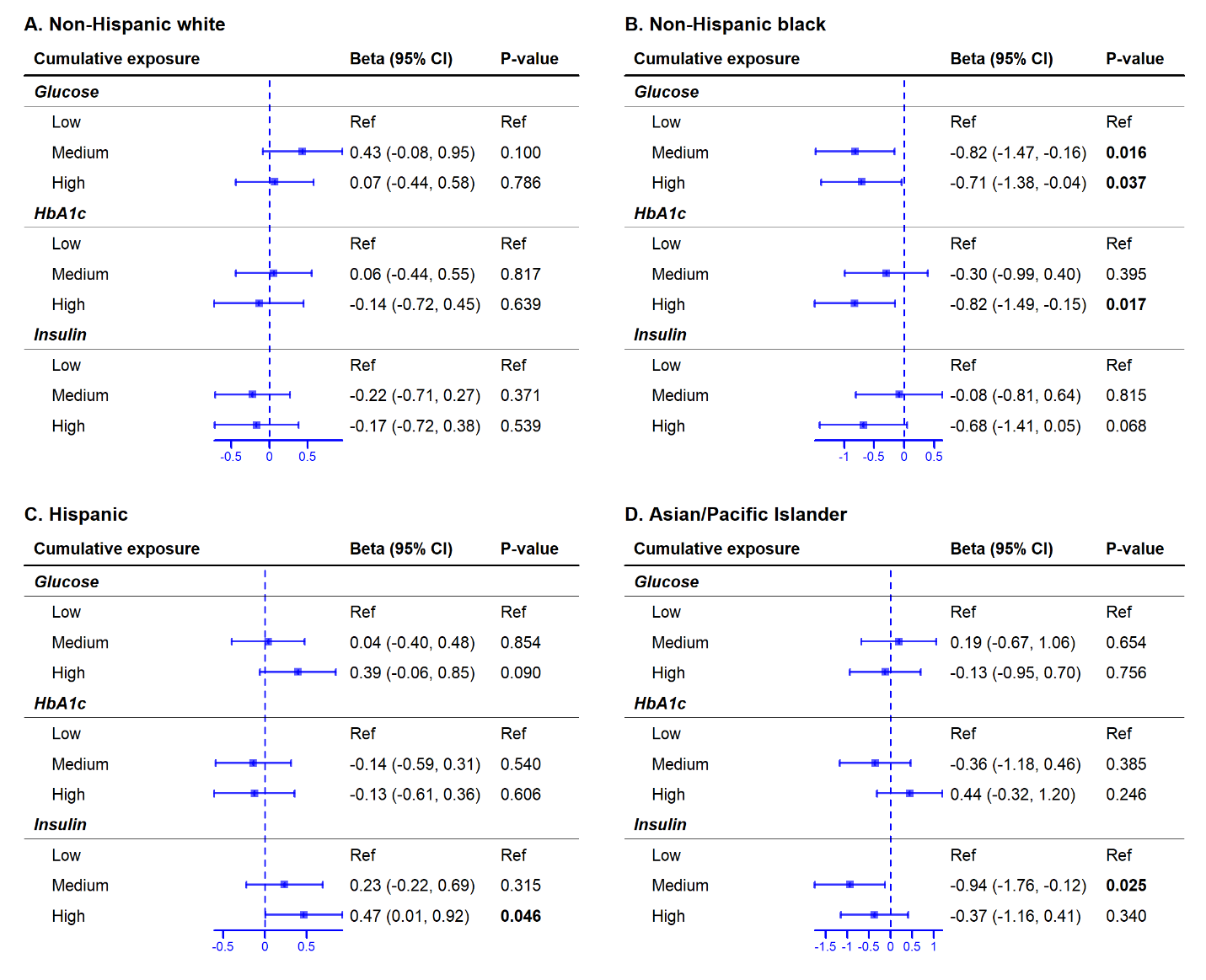


**Figure S4:** Change in PAA associated with glycemic marker changes at each gestational window among women without GDM or preeclampsia by race/ethnicity. Each horizontal line indicates the lower and upper bounds of the 95% CI, and a rectangle on middle of the horizonal line indicates the average change (i.e., beta) in PAA. Broken vertical lines indicate the null hypothesis of no change.


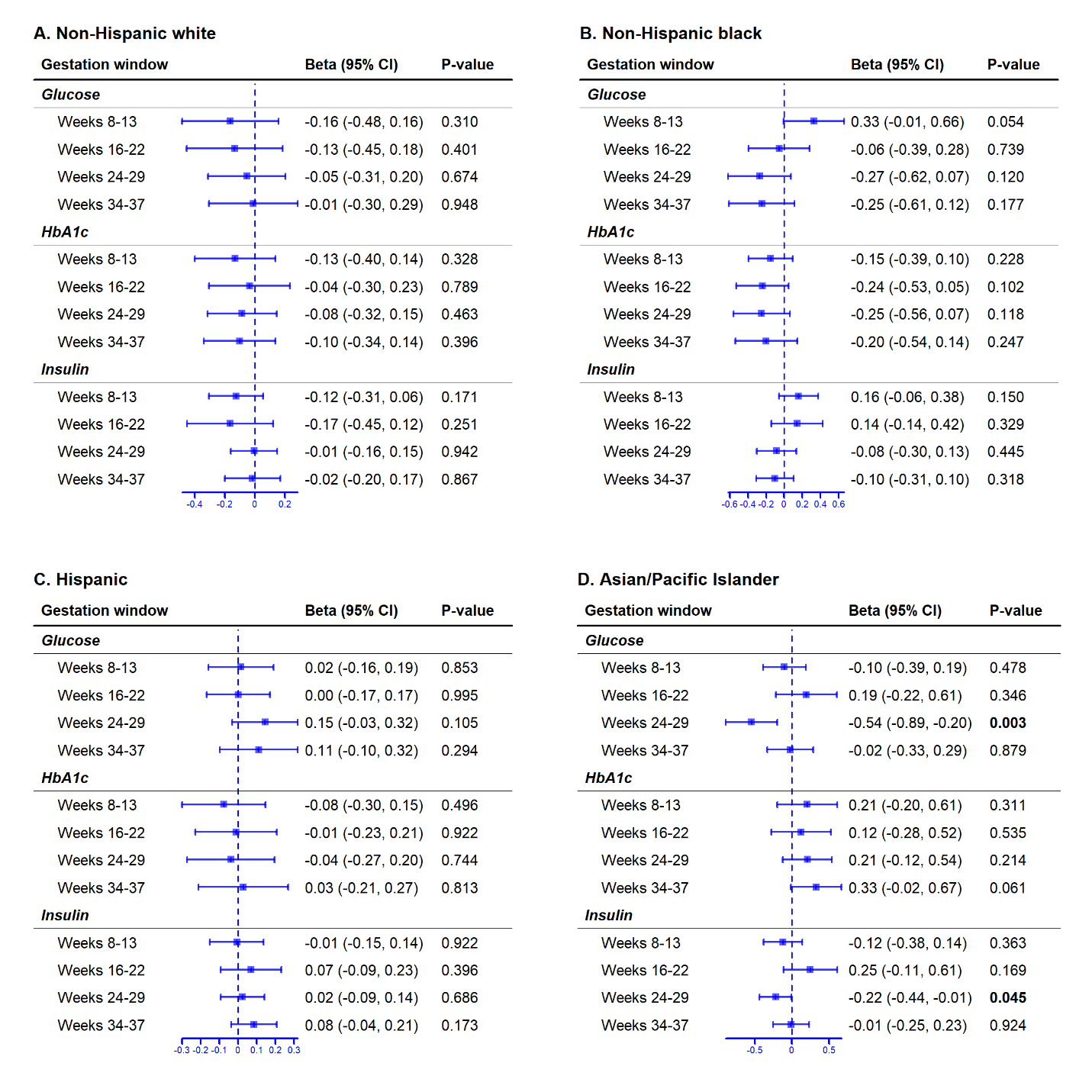


**Figure S5:** Change in PAA associated with changes in glycemic markers at each gestational window among in the full cohort with (left panel) and without (right panel) GDM or preeclampsia (PreE). Low exposure level and low-order trajectory groups are reference groups. Each horizontal line indicates the lower and upper bounds of the 95% CI, and a rectangle in the middle of the horizonal line indicates the average change (i.e., beta) in PAA. Broken vertical lines indicate the null hypothesis of no change.


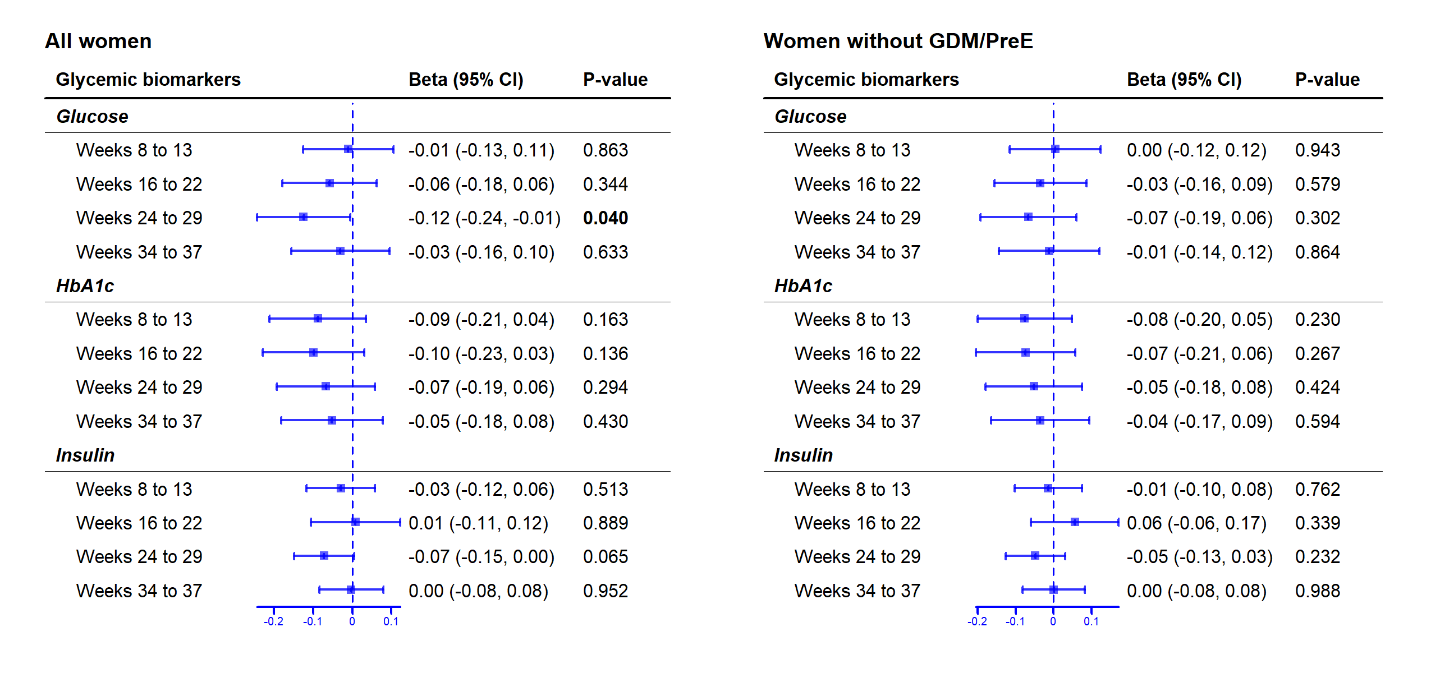


**Figure S6:** Change in PAA associated with glycemic markers trajectory group(s) in the full cohort with (left panel) and without (right panel) GDM or preeclampsia (PreE). Low exposure level and low-order trajectory groups are reference groups. Each horizontal line indicates the lower and upper bounds of the 95% CI, and a rectangle in the middle of the horizonal line indicates the average change (i.e., beta) in PAA. Broken vertical lines indicate the null hypothesis of no change.


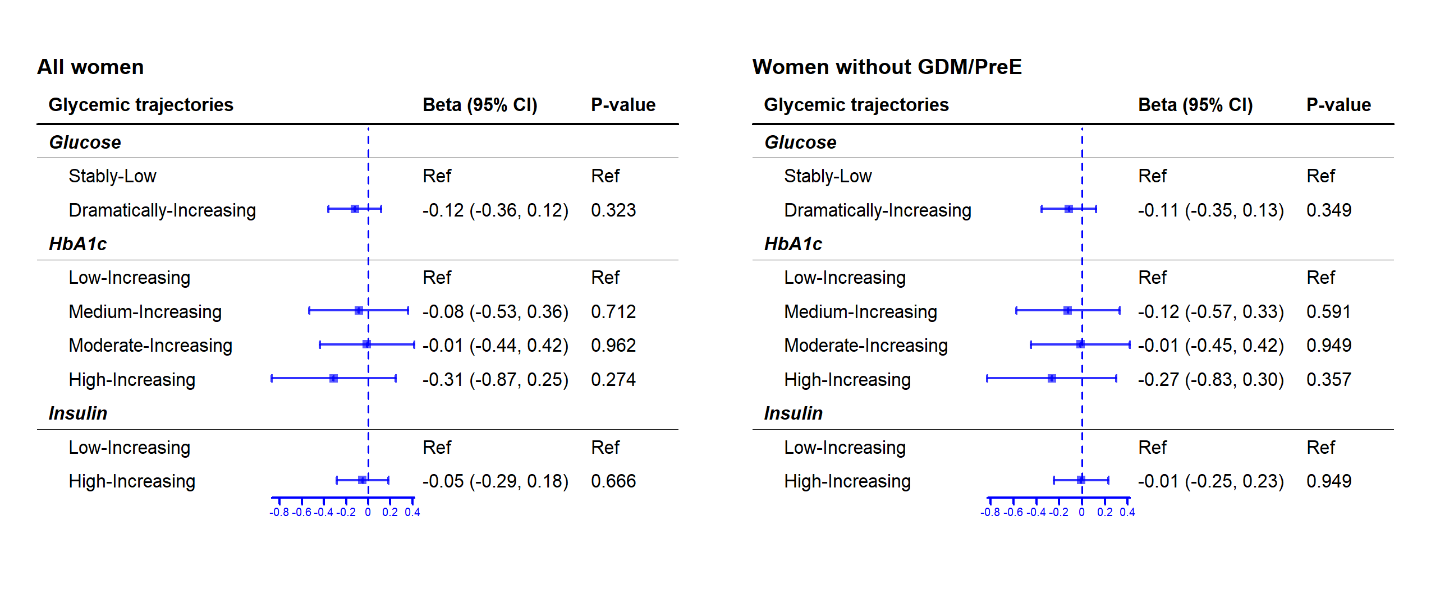


**Figure S7:** Change in PAA associated with glycemic marker trajectory groups among women without GDM or preeclampsia by race/ethnicity. Each horizontal line indicates the lower and upper bounds of the 95% CI, and a rectangle in the middle of the horizontal line indicates the average change (i.e., beta) in PAA. Broken vertical lines indicate the null hypothesis of no change. Lowest-order trajectory group is the reference group for each glycemic marker.


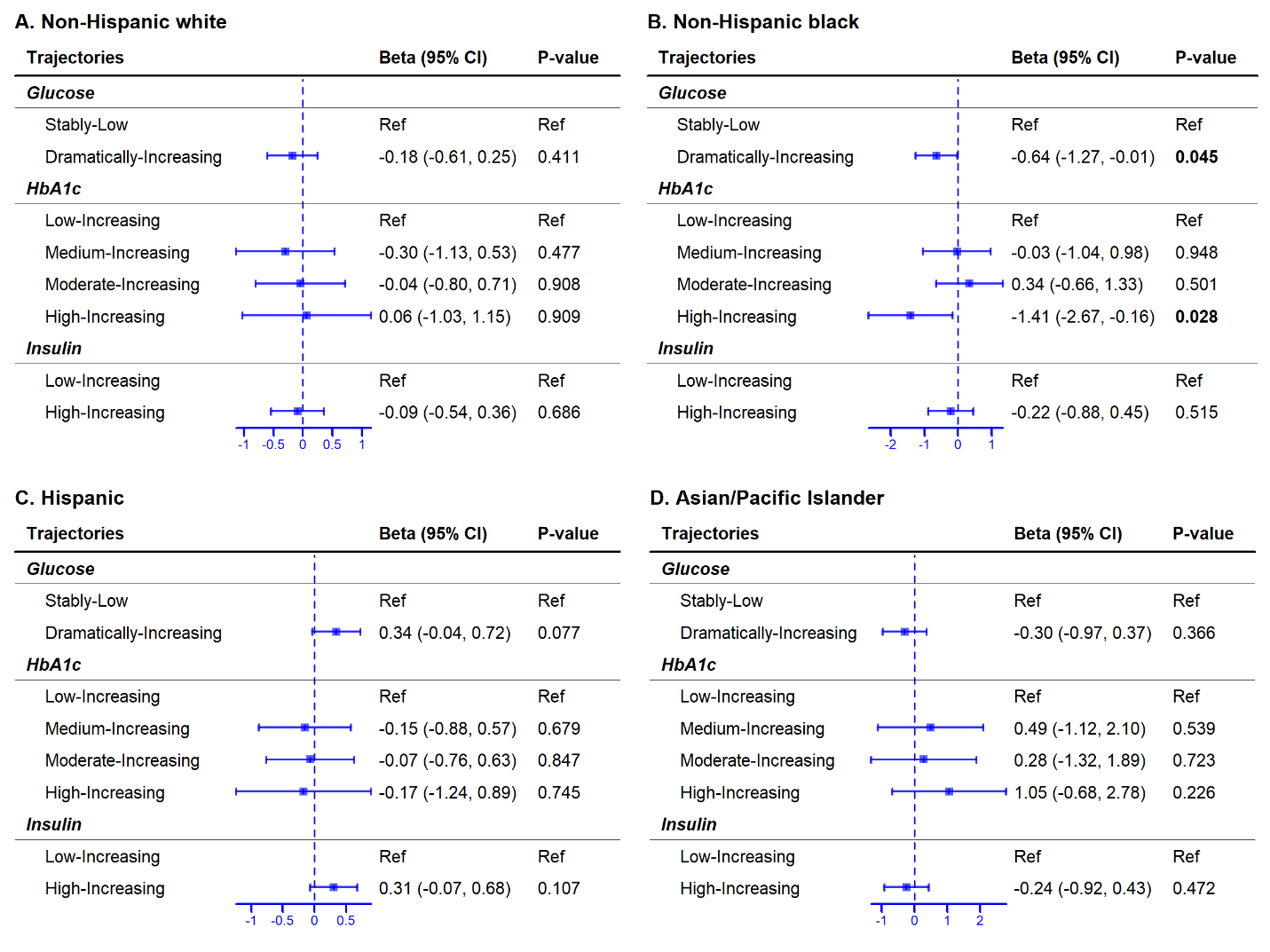

Supplement: Supplementary file 1 — Additional file 1 [file 13148_2025_1825_MOESM1_ESM.docx]
